# Controlling the All-Solid Surface Reaction Between an Li_1.3_Al_0.3_Ti_1.7_(PO_4_)_3_ Electrolyte and Anode Through the Insertion of Ag and Al_2_O_3_ Nano-Interfacial Layers

**DOI:** 10.3390/ma18030609

**Published:** 2025-01-29

**Authors:** Gwanhee Song, Bojoong Kim, Inkook Hwang, Jiwon Kim, Jinmo Kim, Chang-Bun Yoon

**Affiliations:** 1 Department of Advanced Materials Engineering, Tech University of Korea, Siheung-si 15073, Republic of Korea; 2 Korea Photonics Technology Institute (KOPTI), Gwangju 61007, Republic of Korea; jmkim@kopti.re.kr

**Keywords:** solid electrolyte, Li_1+x_Al_x_Ti_2−x_(PO_4_)_3_ (LATP), Ag coating, Al_2_O_3_ coating, atomic layer deposition

## Abstract

Solid-state lithium batteries are considered ideal due to the safety of solid-state electrolytes. The Na superionic conductor-type Li_1.3_Al_0.3_Ti_1.7_(PO_4_)_3_ (LATP) is a solid electrolyte with high ionic conductivity, low cost, and stability. However, LATP is reduced upon contact with metallic lithium, leading to lithium dendrite growth on the anode during charging. In this study, LATP was synthesized, and the relationship between crystallinity and ionic conductivity was investigated at different heat treatment temperatures. Optimal sintering conditions and ionic conductivity were analyzed for sintering temperatures from 800 to 1000 °C. To suppress reactions with Li metal, 50 nm thick Ag and 10 nm thick Al_2_O_3_ layers were deposited on LATP via DC sputtering and plasma-enhanced atomic layer deposition. The electrochemical stability was tested under three conditions: uncoated LATP, Al_2_O_3_-coated LATP, and Ag+Al_2_O_3_-coated LATP. The stability improved in the following order: uncoated < Al_2_O_3_-coated < Ag+Al_2_O_3_-coated. The Al_2_O_3_ coating suppressed secondary phase formation by preventing direct contact between LATP and Li, while Ag coating mitigated charge concentration, inhibiting dendrite growth. These findings demonstrate that Ag and Al_2_O_3_ nano-layers enhance electrolyte stability, advancing solid-state battery reliability and commercialization.

## 1. Introduction

Rechargeable batteries are a crucial element in energy storage and conversion. Among current battery technologies, lithium-based batteries, such as Li-ion batteries (LIBs), are considered the most promising because they exhibit high energy density and stable charging efficiency [1,2,3,4,5]. Conventional Li batteries typically use organic liquid electrolytes (LEs) with relatively high ionic conductivity, which leads to safety concerns, insufficient lifetime, high cost, and low power density. Furthermore, the energy density of liquid-electrode LIBs with current cathode and anode technology is anticipated to reach its maximum value in the near future [6,7,8,9,10].

In this context, current representative approaches with respect to cathode materials include a series of layered Ni-rich lithium transition metal oxides, Li[Ni_1−x−y_Co_x_Al_y_]O_2_ (NCA), and Li[Ni_1−x−y_Co_x_Mn_y_]O_2_ (NCM) [11,12,13,14,15,16], while approaches with respect to the anode material include Li metal, silicon, silicon composite, and silver [17,18,19,20,21,22]. However, most of these improved anode and cathode materials are unstable in liquid electrolytes because of severe side reactions such as electrolyte decomposition, leading to gassing problems after undergoing many cycles. Moreover, dangerous Li dendrites are easily formed in batteries containing LEs, which leads to short circuits and thermal runaway [5,8,9,23,24,25].

All-solid-state Li batteries with nonflammable solid electrolytes can help address some of these safety issues. Compared to liquid-electrolyte Li batteries, all-solid-state batteries are safer and have a longer cycle life, higher energy density, fewer packaging requirements, and state-of-charge monitoring circuits. In recent years, sulfide- and oxide-based all-solid-state batteries have been actively developed to replace liquid batteries. Sulfide electrolytes exhibit excellent ionic conductivities (equal to or greater than most liquid electrolytes) and have attracted increased attention. For example, Li_10_GeP_2_S_12_ exhibits an ionic conductivity of 1.2 × 10^−2^ S cm^−1^, while Li_9.54_Si_1.74_P_1.44_S1_1.7_Cl_0.3_ exhibits a higher ionic conductivity of 2.5 × 10^−2^ S cm^−1^ and has been demonstrated in all-solid-state batteries (SSBs) [22,26,27,28,29]. However, in sulfide-based all-solid-state batteries, toxic H_2_S gas is generated when reacting with water in the air, making mass production difficult.

In contrast, oxide-based SSBs show excellent stability in air despite the difficulty involved in sintering. Oxide-based electrolytes include Li superionic conductor (LISICON) oxides, Na superionic conductor (NASICON) oxides, perovskite-type oxides, garnet-type oxides, and glass/glass ceramic/crystalline electrolytes [30,31,32,33,34,35,36,37]. Some of these oxide-based electrolytes exhibit high ionic conductivities at 25 °C (close to 1 mS cm^−1^) that are comparable to those of liquid electrolytes [25,38,39,40]. Among them, the NASICON-type electrolyte Li_1.3_Al_0.3_Ti_1.7_(PO_4_)_3_ (LATP) has received considerable attention after its initial development in the 1990s by Anno et al. LATP exhibits an ionic conductivity of 0.1 mS cm^−1^ at room temperature due to the doping of Al in the lattice. The limitations of LATP include the formation of dendrites at the interface with the Li anode and the reduction of Ti^4+^ to Ti^3+^, and further research is necessary to overcome these limitations [41,42,43,44,45,46,47,48].

In this study, LATP solid electrolytes were synthesized using raw powders through conventional mechanical milling. The mixed powders were calcined at 850 °C for 2 h, followed by an additional 24 h of ball milling to obtain fine powders. The powders were then heat-treated at sintering temperatures ranging from 800 to 1000 °C. The relationship between crystallinity and ionic conductivity was investigated by measuring the crystallinity, density, and grain size. To mitigate the reactions between lithium ions and the solid electrolyte, the LATP surfaces were coated with 50 nm thick Ag and 10 nm thick Al_2_O_3_ layers using DC sputtering and plasma-enhanced atomic layer deposition (PE-ALD), respectively. To evaluate the interfacial reactivity, samples were prepared as follows: bare LATP, LATP coated on both sides with Al_2_O_3_ nano-layers, and LATP coated on both sides with Al_2_O_3_ and Ag. Lithium metal was attached to both sides of each sample, and DC cycling tests were performed. The interfacial reactivity followed the order of bare LATP, Al_2_O_3_-coated LATP, and LATP with Al_2_O_3_ and Ag coatings and showed a progressive reduction in interfacial reactions. In conclusion, both Al_2_O_3_ coatings and Ag/Al_2_O_3_ composite coatings effectively reduced the reaction between LATP and lithium ions, suppressed lithium dendrite growth, and enhanced the electrochemical stability of the solid electrolyte [41,49,50].

## 2. Materials and Methods

### 2.1. Synthesis and Sintering of Li_1.3_Al_0.3_Ti_1.7_(PO4)_3_ (LATP)

Li_2_CO_3_ (99%, Sigma-Aldrich, Seoul, Republic of Korea), Al_2_O_3_ (99%, Sigma-Aldrich), TiO_2_ (99%, Sigma-Aldrich), and NH_4_H_2_PO_4_ (99%, Sigma-Aldrich) were used as the starting materials for the LATP synthesis. LATP was synthesized via a typical wet solid-state reaction method. The raw materials were weighed according to the stoichiometric ratios of LATP and mixed via ball milling at 200 rpm for 4 h. Alcohol was used as the solvent for mixing, and zirconia balls were added to ensure homogeneous mixing. The mixture was placed in an alumina crucible and calcined at 850 °C for 2 h to synthesize the LATP (NASICON) phase. After heat treatment, the agglomerated powder clumps were crushed using a mortar and pestle, followed by further grinding with zirconia balls in a ball mill at 200 rpm for 24 h. The resulting powder was sieved through a fine mesh to obtain uniformly fine LATP powder. The synthesized LATP powder was uniaxially pressed into 1.0 mm thick pellets using a 10 mm mold. To enhance green density, the pellets were subjected to cold isostatic pressing (SCIP50150-3 KB, Samyang Ceratech, Incheon, Republic of Korea). Finally, the pellets were sintered in an alumina crucible at 800–1000 °C for 6 h to produce high-density polycrystalline sintered bodies. To prevent contamination from the crucible and suppress the volatilization of lithium during heat treatment, LATP powder was sprinkled below the formed body, and the pellet was covered with additional powder before heat treatment. The sintered LATP pellets were polished to a thickness of 0.5 mm using sandpapers ranging from #180 to #2000 mesh to remove surface contamination.

### 2.2. Nano-Al_2_O_3_ and Ag Layer Coating Methods

Al_2_O_3_ was coated on LATP via PE-ALD (iOV dx2, iSAC Research, Daejeon, Republic of Korea) at 250 °C. Trimethylaluminum (iChems, Gyeonggi-do, Republic of Korea) was used as the Al_2_O_3_ precursor, and O_2_ was used as the reactant under plasma. Argon was used as both the carrier and purge gas. The pressure inside the PE-ALD chamber was maintained at 1.1–1.4 Torr during plasma application, with an RF plasma power of 200 W. Al_2_O_3_ was deposited through repeated cycles of gas injection, purging, oxidation, plasma discharge, and purging for 110 cycles. This resulted in a thin film with a growth per cycle of 0.9 Å, leading to a total thickness of 10 nm. Ag thin films were deposited using a DC magnetron sputtering system (DC-Sputter, BLS, Pyeongtaek-si, Gyeonggi-do, Republic of Korea). A high-quality Ag thin film with lithium affinity was deposited as follows. The sputtering chamber pressure was reduced to 1 × 10^−5^ Torr using a high-performance turbo pump connected to a rotary pump. Argon gas was injected at 10 sccm for 30 s at a pressure of 0.15 Torr. The power and deposition time were set to 40 W and 15 s, respectively. A Ag thin film with a thickness of 50 nm was deposited.

### 2.3. Battery Performance Evaluation of Thin-Film Coated LATP Solid-State Electrolyte

Au was deposited on both sides of the LATP solid-state electrolyte to fabricate an Au/LATP solid electrolyte/Au structure; this structure was used in electrochemical testing. The charge–discharge stability was investigated as follows. The LATP sintered body was uniformly processed to a thickness of 500 μm, and Li plates were placed on both sides. The samples were bonded using the CIP process and assembled into 2032 coin cells. The electrochemical performance was evaluated via DC cycling at a constant current of 10 μA. The electrochemical cycling tests were conducted using a battery charge–discharge testing system (WBCS 3000 Cycler, WonATech, Seoul, Republic of Korea) with precise control of electrochemical processes. All electrochemical properties and charge–discharge characteristics were evaluated without applying any additional external pressure after coin-cell fabrication. An electrochemical impedance spectrometer (EIS, VersaSTAT 3, Princeton Applied Research, Oak Ridge, TN, USA) over a frequency range of 100 Hz to 100 kHz with a voltage amplitude of 5 mV was used for impedance measurements. The crystal structure of the solid electrolyte and its composites with the cathode and anode was analyzed via X-ray diffraction (XRD) analysis (D2 PHASER, Bruker, Billerica, MA, USA). The surface morphologies of the solid electrolyte, anode, and cathode were observed using a scanning electron microscope (Nova NanoSEM 450, FEI, Hillsboro, OR, USA).

## 3. Results and Discussion

### 3.1. Synthesis of LATP Solid Electrolyte and Structural Characterization

**Figure 1:**
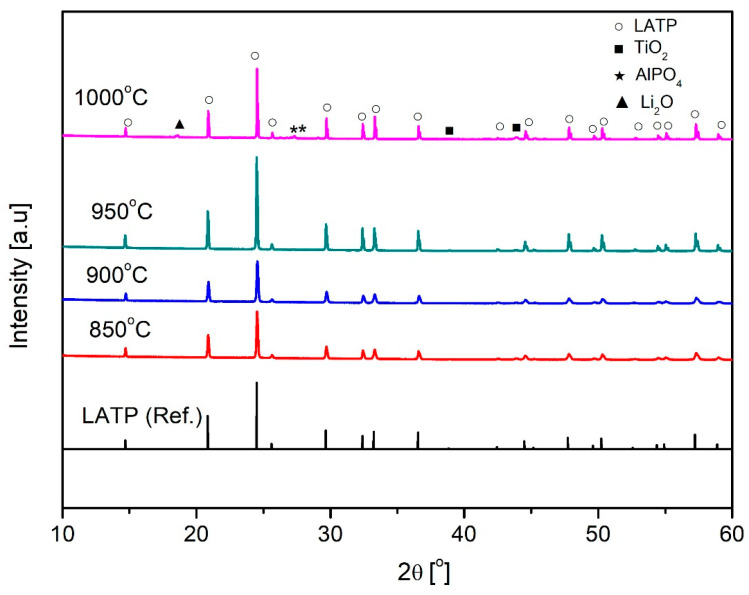
X-ray diffraction patterns of Li_1.3_Al_0.3_Ti_1.7_(PO4)_3_ (LATP) at different sintering temperatures (850 °C to 1000 °C).

Figure 1 shows the XRD patterns of the synthesized LATP solid electrolyte at different temperatures, with the data measured across the entire range from 10° to 60°. The synthesized LATP sintered powder exhibited typical XRD peaks of the NASICON structure in the calcination temperature range of 850 to 1000 °C. No secondary phase was detected at 950 °C. However, a secondary phase with peaks appeared at 1000 °C. This secondary phase indicates a transformation of LATP due to the volatilization of Li, resulting in TiO_2_, AlPO_4_, and Li_2_O phases [44,50,51,52]. The LATP-sintered body, heat-treated at 950 °C, exhibited a pure NASICON structure free of secondary phases.

**Figure 2:**
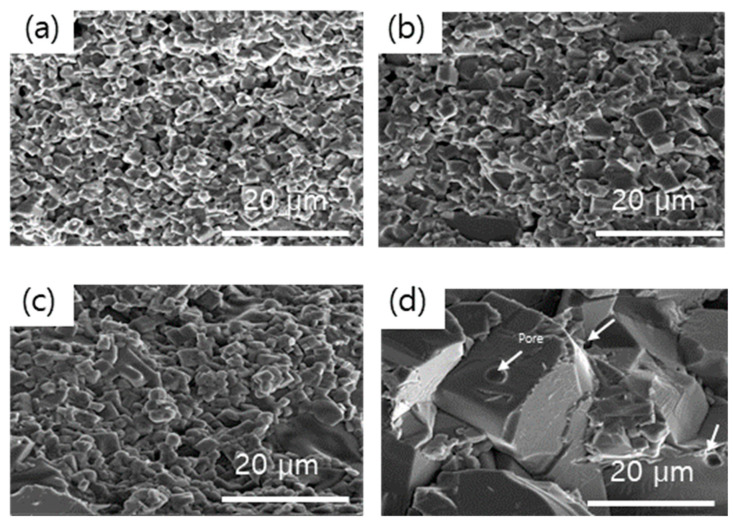
Cross-sectional scanning electron microscopy (SEM) images of the LATP solid electrolytes at different temperatures, (**a**) 850 °C, (**b**) 900 °C, (**c**) 950 °C, and (**d**) 1000 °C, for a sintering time of 6 h.

Figure 2 shows the scanning electron microscopy (SEM) images of the synthesized LATP solid electrolytes. Figure 2a–d display the cross-sectional images of the samples sintered at 850, 900, 950, and 1000 °C for 6 h, respectively. The grain size was approximately 2.0 µm at 850 °C and increased with an increase in the sintering temperature, reaching 3.8 µm at 900 °C, 6.3 µm at 950 °C, and 15 µm at 1000 °C. This indicates a significant increase in the sintering-driving force around 1000 °C, which, in turn, promotes material diffusion and grain growth. Square-shaped grains, characteristic of LATP, were observed. These results are in good agreement with those reported in previous studies [51,52,53]. At a heat treatment temperature of 1000 °C, a significant number of pores were observed at the grain boundaries as grain growth and densification progressed, consistent with the XRD results indicating the formation of secondary phases due to Li volatilization.

**Figure 3:**
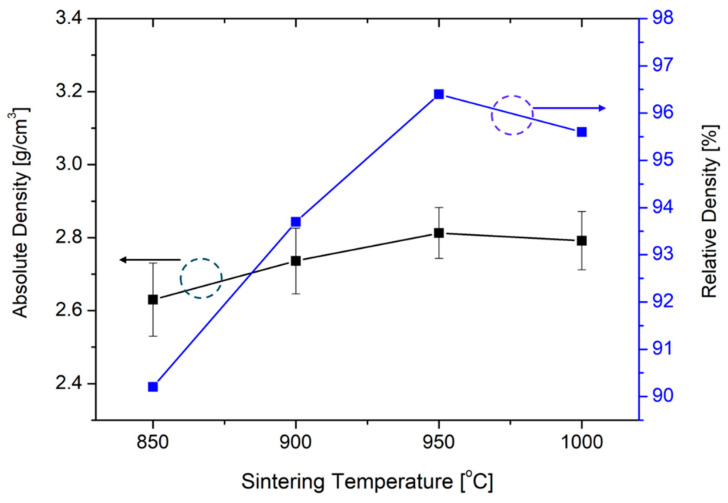
Measured absolute and relative densities of LATP as a function of the sintering temperature.

Figure 3 shows the measured absolute and relative densities of LATP as a function of the sintering temperature. The theoretical density of LATP at room temperature was set to 2.92 g/cm^3^, and the absolute density was measured using the Archimedes method. The relative density was calculated by dividing the absolute density by the theoretical density. The density at 850 °C was 2.63 g/cm³ (90.2%). With an increase in the temperature, the density first increased, reaching 2.81 g/cm^3^ (96.2%) at 950 °C, and then decreased to 2.79 g/cm^3^ (95.6%) at 1000 °C. Similarly to that in the XRD and SEM results, the observed initial densification with increasing temperature is probably due to grain growth, whereas the decrease at higher temperatures is attributed to pore formation resulting from lithium volatilization.

**Figure 4:**
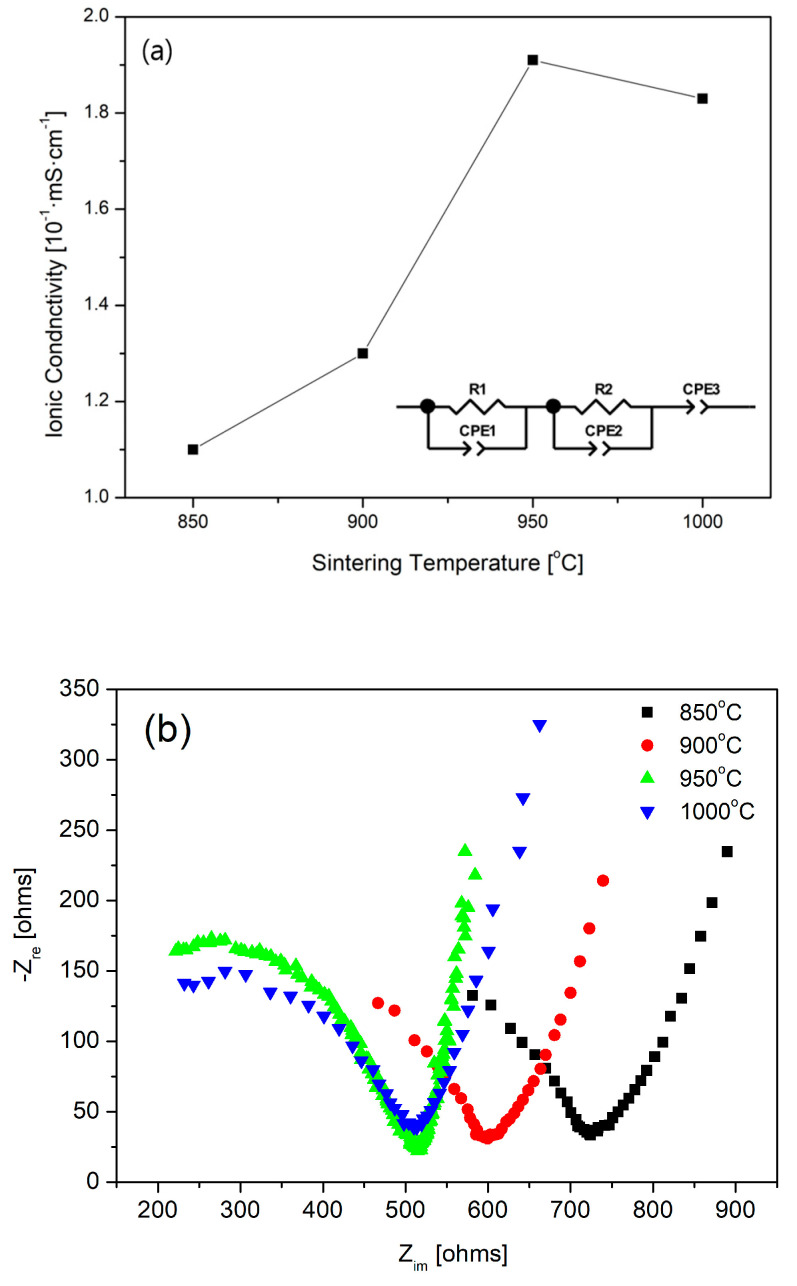
Ionic conductivity of LATP at different sintering temperatures: (**a**) conductivity graph; (**b**) temperature-specific data.

Figure 4 shows the AC impedance of the LATP sintered bodies measured at 25 °C as a function of the sintering temperature. A typical Nyquist plot was observed when lithium metal was attached to both sides of the LATP sintered body. The Nyquist plot consists of two semicircles and a straight line, which were analyzed using the equivalent circuit shown in the inset of Figure 4a. Each semicircle corresponds to a resistor (R) and a constant-phase element (CPE) connected in parallel. R1 and CPE1, respectively, denote the resistance and capacitance attributed to the bulk; R2 and CPE2, respectively, denote the resistance and capacitance attributed to the grain boundaries. CPE3 denotes the capacitance at the interface. The ionic conductivities measured from the equivalent circuit are plotted against the sintering temperature in Figure 4b. The ionic conductivity was the maximum with a value of 1.9 × 10^−1^ mS cm⁻¹at 950 °C. At 1000 °C, the ionic conductivity decreased owing to lithium volatilization. As shown in the SEM image of grain boundaries (Figure 2) and the XRD results indicating the formation of secondary phases (Figure 1), the increased impedance is attributable to the formation of voids and secondary phases. Based on density, ionic conductivity, and XRD analysis, the optimal heat treatment temperature was determined to be 950 °C [54].

**Figure 5:**
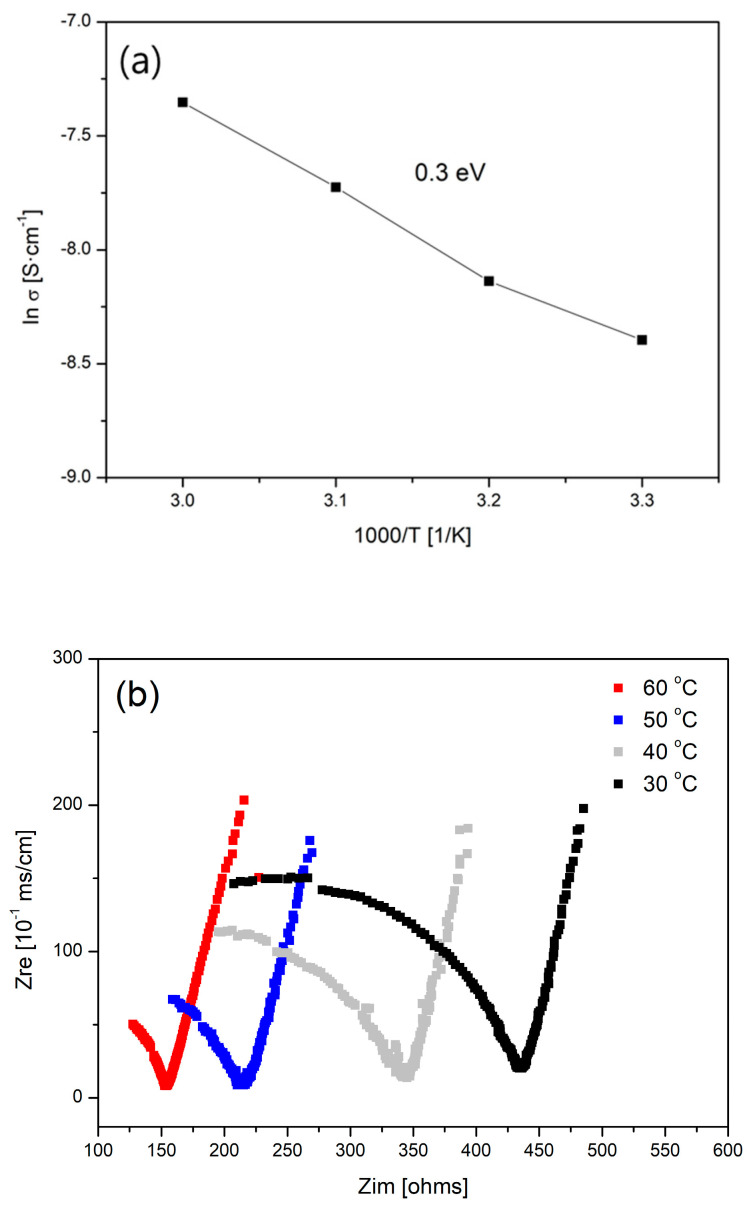
Activation energy measurements for LATP sintered at 950 °C: (**a**) ln σ vs. 1000/T plot graph; (**b**) Nyquist plot results at different temperatures.

The activation energy (E_a_) of LATP was derived from the results of the ionic conductivity measurements at various temperatures, as shown in Figure 5a. The temperature was varied from 30 °C to 60 °C, and the logarithm of the conductivity was plotted against 1/T. The logarithmic form of the Arrhenius equation (Equation (1)) indicates that the negative slope of the plot corresponds to the activation energy as follows:

σ_T_ = A × exp(−E_a_/kT) (1)

where σ is the ionic conductivity, T is the absolute temperature, A is the pre-exponential constant, E_a_ is the activation energy for ionic conductivity, and k is the Boltzmann constant. The resistance of LATP was inversely proportional to the temperature, as shown in Figure 5b; the resistance decreased from 440 Ω to 160 Ω. The measured activation energy was 0.3 eV, which is a low activation energy. These results demonstrate the excellent performance of LATP as a solid electrolyte [55].

### 3.2. Evaluation of Electrochemical Stability of Solid Electrolytes

**Figure 6:**
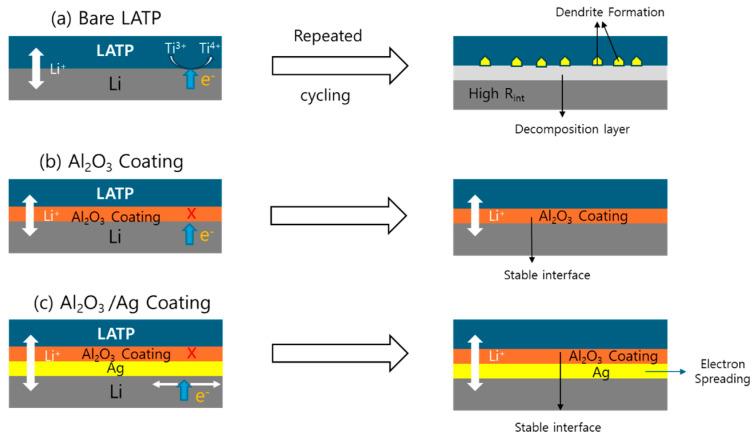
Mechanisms observed in LATP-based all-solid-state batteries during charge–discharge cycles: (**a**) formation of secondary phases and dendrites, (**b**) effects of Al_2_O_3_ nano-layer coating, and (**c**) effects of Ag/Al_2_O_3_ nano-layer coating.

To reduce the reaction between lithium ions and the solid electrolyte, the LATP surfaces were coated with a 50 nm thick Ag layer and a 10 nm thick Al_2_O_3_ layer using DC sputtering and PE-ALD, respectively. DC cycling tests were performed on the following samples: bare LATP, LATP coated with Al_2_O_3_ nanolayers on both sides, and LATP coated with both Al_2_O_3_ and Ag on both sides. Lithium metal was attached to both sides of each sample. The mechanisms associated with the roles of each nanolayer are illustrated in Figure 6.

As shown in Figure 6a, when LATP forms an interface with Li metal during the charge–discharge processes, the movement of electrons results in the reduction of Ti⁴⁺ to Ti^3+^, leading to the formation of a secondary phase. Simultaneously, the migration of Li⁺ ions results in the formation of dendrites and the development of cracks. To address this issue, the interface between LATP and Li was coated with Al_2_O_3_. Figure 6b illustrates the mechanism by which PE-ALD is applied to coat the LATP interface with Al_2_O_3_. The application of the coating suppresses electron migration while allowing for the passage of Li⁺ ions, thereby mitigating the formation of secondary phases resulting from Ti reduction [41,56]. The Al_2_O_3_ coating, with its excellent hardness, acts as a physical barrier that inhibits dendrite formation and serves as a protective layer that prevents further reduction of Ti in LATP. In uncoated LATP, Li dendrites are formed, Ti reduction progressively penetrates into the bulk region, and cracks develop, ultimately leading to mechanical failure.

As shown in Figure 6c, when a 50 nm Ag layer is deposited on a 10 nm Al_2_O_3_ layer, the Al_2_O_3_ coating suppresses electron migration, and the Ag layer functions as an electron-spreading layer. This configuration effectively mitigates charge concentration due to Li-ion migration and, thus, eliminates dendrite formation at the fundamental level. The stability of the solid-state electrolyte and coating layers in contact with lithium metal was investigated using density functional theory (DFT) calculations [50]. The adsorption energy of a single lithium atom in LATP and Al_2_O_3_ were calculated using DFT. The adsorption energy of a single lithium atom in LATP was −2.88 eV, a highly negative value indicating the instability of LATP with respect to lithium metal. In contrast, the calculated adsorption energy of a single lithium atom in Al_2_O_3_ was +3.17 eV, with the highly positive value indicating the high stability of Al_2_O_3_ against lithium metal. These results suggest that Al_2_O_3_ is electrochemically stable. Coating LATP with Al_2_O_3_ reduces the HOMO energy level of LATP, thereby increasing its resistance to oxidation. Additionally, the LUMO energy level is elevated, suppressing reduction reactions on the LATP surface and enhancing its electrochemical stability. Consequently, coating LATP with a nanoscale Al_2_O_3_ layer increases the gap between the HOMO and LUMO energy levels, ultimately improving the overall electrochemical stability of LATP.

**Figure 7:**
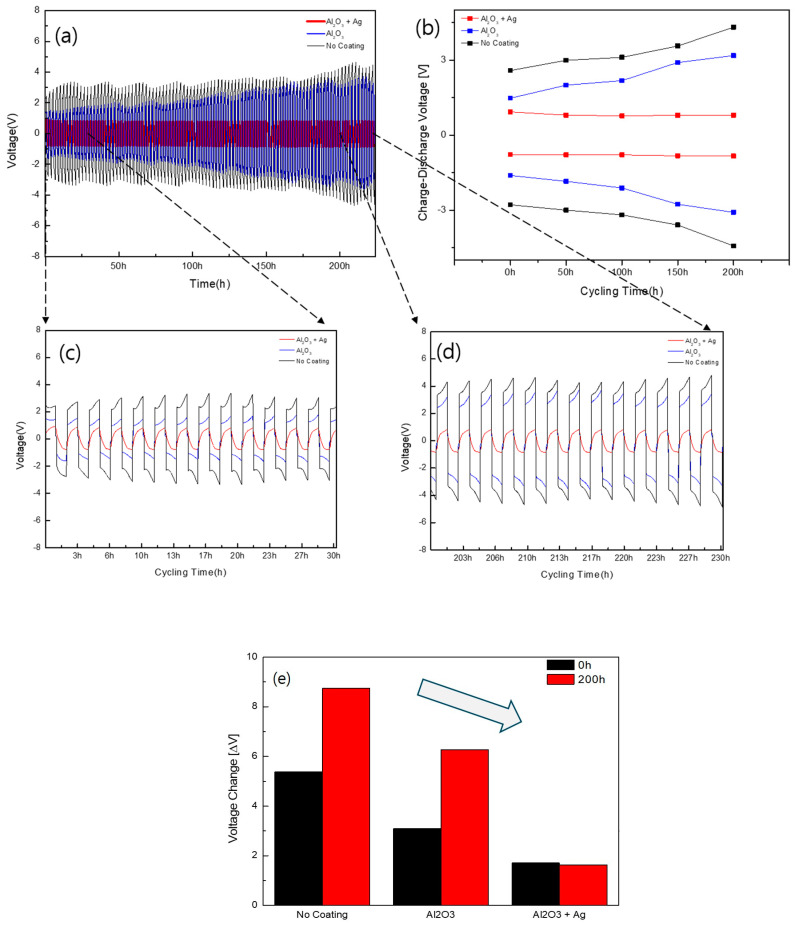
Performance evaluation of Li–Li symmetric cells with LATP sintered samples: bare LATP, 10 nm Al_2_O_3_-coated sample, and 10 nm Al_2_O_3_/50 nm Ag-coated sample. (**a**) Full DC cycling graph. (**b**) Maximum and minimum voltage change graph. (**c**) Charge–discharge profile at 0 h (**d**) Charge–discharge profile at 200 h. (**e**) Voltage change (Vmax−Vmin) after 200 h for each coating type.

The long-term interfacial stability between Li metal and solid electrolytes was evaluated via DC cycling measurements. Figure 7 presents the results of the DC cycling measurements for the Li–Li symmetric cell. Three samples—bare LATP solid electrolyte (no coating), LATP coated with a 10 nm Al_2_O_3_ layer via PE-ALD (Al_2_O_3_), and LATP coated with 10 nm Al_2_O_3_ and 50 nm Ag layers (Al_2_O_3_ + Ag)—were subjected to DC cycling for over 200 h, and these results were compared. As shown in Figure 7a, the bare LATP exhibited a high initial voltage and a continuous voltage increase over 200 h. In contrast, the sample with 10 nm Al_2_O_3_ showed a marginal increase in voltage compared to the initial cycles, whereas the sample with 10 nm Al_2_O_3_ and 50 nm Ag showed stable behavior with no voltage change, maintaining a stable DC cycling performance below 1 V. Figure 7b illustrates the maximum and minimum voltages during the charge–discharge cycles from 0 to 200 h. The bare LATP and Al_2_O_3_-coated samples showed increasing maximum and minimum voltages over cycles. However, the sample with Al_2_O_3_ + Ag showed no voltage fluctuation, indicating stable performance. Figure 7c,d depict the charge–discharge profiles at 0 and 200 h, respectively. The driving voltage increased progressively in the later cycles for the bare LATP, 10 nm Al_2_O_3_, and 10 nm Al_2_O_3_/50 nm Ag samples. The increase in the driving voltage was in the following order: bare LATP > 10 nm Al_2_O_3_ > 10 nm Al_2_O_3_/50 nm Ag. The Al_2_O_3_ + Ag-coated sample exhibited no changes in the charge–discharge profiles, suggesting stability during cycling. Figure 7e shows the voltage changes before and after cycling. The results demonstrate that the LATP sample coated with 10 nm Al_2_O_3_ and 50 nm Ag exhibits the most stable behavior, with no change observed below 2 V. The Al_2_O_3_ layer deposited via ALD effectively suppressed the formation of secondary phases on the LATP surface by preventing direct contact between LATP and Li. The Ag layer further improved the stability by distributing charge at the interface and inhibiting dendrite formation [41,57,58,59].

**Figure 8:**
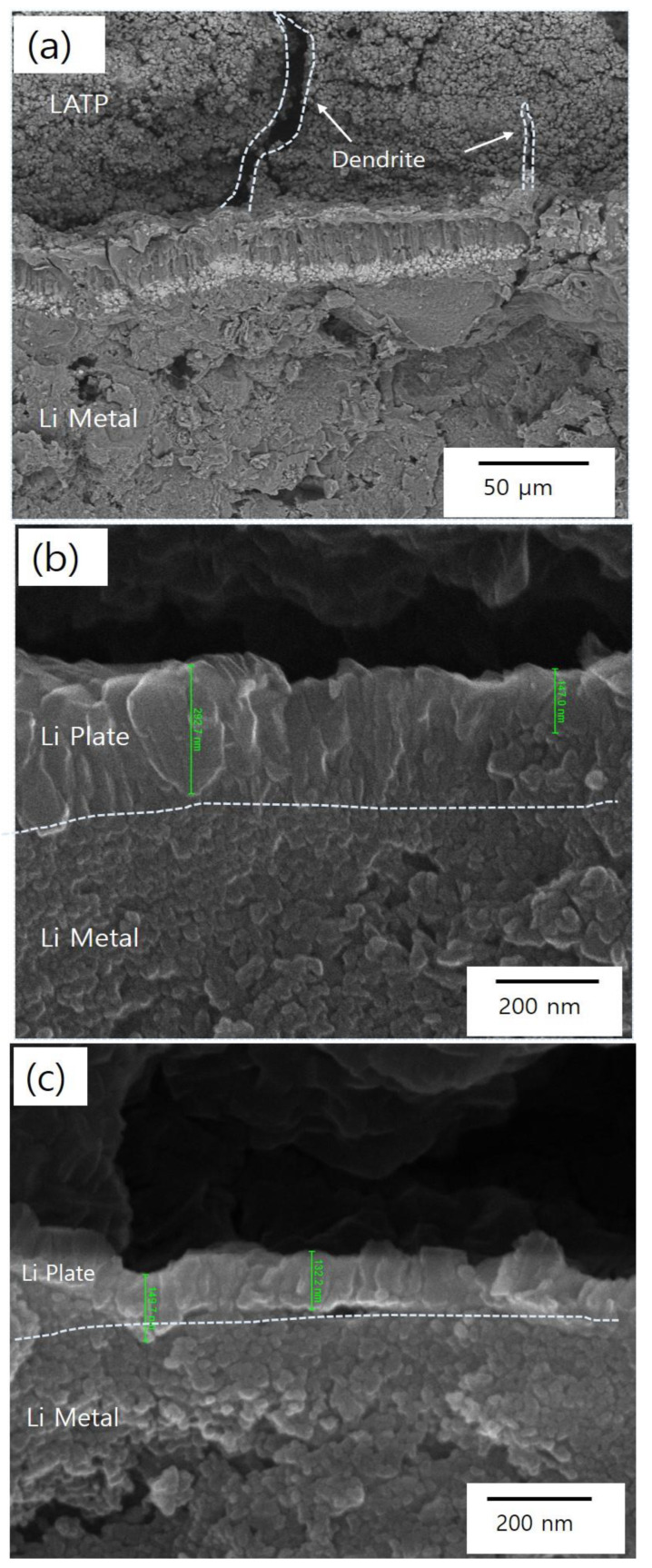
Cross-sectional analysis after 200 h of DC cycling: (**a**) LATP only, (**b**) Al_2_O_3_-coated LATP, and (**c**) Al_2_O_3_/Ag-coated LATP.

Figure 8 shows SEM images of cross-sections from samples after 200 h of DC cycling. Figure 8a shows the formation of dendrites from the Li metal penetrating the LATP, leading to microcrack development within the LATP. Figure 8b depicts LATP coated with a 10 nm Al_2_O_3_ layer, where lithium is deposited in a plate-like form on the Li-metal side. Figure 8c demonstrates the results for LATP coated with a 10 nm Al_2_O_3_ and an additional 50 nm Ag layer. In this case, lithium is also deposited in a plate-like form on the Li-metal side, but, unlike the sample with Al_2_O_3_ coating alone, a highly dense layer approximately 100 nm thick is formed. This dense layer has been demonstrated to be highly effective in preventing dendrite formation.

## 4. Conclusions

In this study, LATP was synthesized using the solid-state reaction method, and the relationship between the crystallinity and ionic conductivity of LATP was experimentally investigated as a function of sintering temperature within the range of 800–1000 °C. The highest density and ionic conductivity (1.9 × 10^−1^ mS cm^−1^) were observed at 950 °C. At temperatures above 1000 °C, micropores caused by lithium volatilization were observed, along with significant grain size increases, decreasing ionic conductivity. The crystallinity, density, and grain size of the LATP solid electrolyte were confirmed to be closely related to the heat treatment temperature. The surface of the LATP pellet was coated with 50 nm thick Ag and 10 nm thick Al_2_O_3_ layers via DC sputtering and PE-ALD, respectively. The nano Al_2_O_3_ and Ag/Al_2_O_3_ coatings effectively reduced the reaction between LATP and lithium ions and suppressed the growth of lithium dendrites. This surface coating strategy is expected to expand the electrochemical stability window of all-solid-state batteries and significantly enhance their commercialization prospects.

## Data Availability

The data presented in this study are available on request from the corresponding author.

## References

[1] Armand M., Tarascon J.M. (2008). Building better batteries. Nature.

[2] Turcheniuk K., Bondarev D., Amatucci G.G., Yushin G. (2021). Battery materials for low-cost electric transportation. Mater. Today.

[3] Goodenough J.B., Park K.-S. (2013). The Li-ion rechargeable battery: A perspective. J. Am. Chem. Soc..

[4] Schmuch R., Wagner R., Hörpel G., Placke T., Winter M. (2018). Performance and cost of materials for lithium-based rechargeable automotive batteries. Nat. Energy.

[5] Hubble D., Brown D.E., Zhao Y., Fang C., Lau J., McCloskey B.D., Liu G. (2022). Liquid electrolyte development for low-temperature lithium-ion batteries. Energy Environ. Sci..

[6] Monroe C., Newman J. (2005). The impact of elastic deformation on deposition kinetics at lithium/polymer interfaces. J. Electrochem. Soc..

[7] Henriksen M., Vaagsaether K., Lundberg J., Forseth S., Bjerketvedt D. (2021). Laminar burning velocity of gases vented from failed Li-ion batteries. J. Power Sources.

[8] Roth E.P., Orendorff C.J. (2012). How electrolytes influence battery safety. Electrochem. Soc. Interface.

[9] Duffner F., Kronemeyer N., Tübke J., Leker J., Winter M., Schmuch R. (2021). Post-lithium-ion battery cell production and its compatibility with lithium-ion cell production infrastructure. Nat. Energy.

[10] Janek J., Zeier W.G. (2016). A solid future for battery development. Nat. Energy.

[11] Julien C.M., Mauger A., Zaghib K., Groult H. (2014). Comparative issues of cathode materials for Li-ion batteries. Inorganics.

[12] Hausbrand R., Cherkashinin G., Ehrenberg H., Gröting M., Albe K., Hess C., Jaegermann W. (2015). Fundamental degradation mechanisms of layered oxide Li-ion battery cathode materials: Methodology, insights and novel approaches. Mater. Sci. Eng. B.

[13] Myung S.-T., Maglia F., Park K.-J., Yoon C.S., Lamp P., Kim S.-J., Sun Y.-K. (2017). Nickel-rich layered cathode materials for automotive lithium-ion batteries: Achievements and perspectives. ACS Energy Lett..

[14] Kim U.-H., Kuo L.-Y., Kaghazchi P., Yoon C.S., Sun Y.-K. (2019). Quaternary layered Ni-Rich NCMA cathode for lithium-ion batteries. ACS Energy Lett..

[15] Zhou P., Meng H., Zhang Z., Chen C., Lu Y., Cao J., Cheng F., Chen J. (2017). Stable layered Ni-Rich LiNi_0.9_Co_0.07_Al_0.03_O_2_ microspheres assembled with nanoparticles as high-performance cathode materials for lithium-ion batteries. J. Mater. Chem. A.

[16] Liu Z., Yu A., Lee J.Y. (1999). Synthesis and characterization of LiNi_1−x−y_Co_x_Mn_y_O_2_ as the cathode materials of secondary lithium batteries. J. Power Sources.

[17] Jin Y., Zhu B., Lu Z., Liu N., Zhu J. (2017). Challenges and recent progress in the development of si anodes for lithium-ion battery. Adv. Energy Mater..

[18] Kim J.-S., Yoon G., Kim S., Sugata S., Yashiro N., Suzuki S., Lee M.-J., Kim R., Badding M., Song Z. (2023). Surface engineering of inorganic solid-state electrolytes via interlayers strategy for developing long-cycling quasi-all-solid-state lithium batteries. Nat. Commun..

[19] Lee Y.-G., Fujiki S., Jung C., Suzuki N., Yashiro N., Omoda R., Ko D.-S., Shiratsuchi T., Sugimoto T., Ryu S. (2020). High-energy long-cycling all-solid-state lithium metal batteries enabled by silver-carbon composite anodes. Nat. Energy.

[20] Franco Gonzalez A., Yang N.-H., Liu R.-S. (2017). Silicon anode design for lithium-ion batteries: Progress and perspectives. J. Phys. Chem. C.

[21] Yang Y., Yuan W., Kang W., Ye Y., Pan Q., Zhang X., Ke Y., Wang C., Qiu Z., Tang Y. (2020). A review on silicon nanowire-based anodes for next-generation high-performance lithium-ion batteries from a material-based perspective. Sustain. Energy Fuels.

[22] Tan D.H.S., Chen Y.-T., Yang H., Bao W., Sreenarayanan B., Doux J.-M., Li W., Lu B., Ham S.-Y., Sayahpour B. (2021). Carbon-free high-loading silicon anodes enabled by sulfide solid electrolytes. Science.

[23] Bachman J.C., Muy S., Grimaud A., Chang H.-H., Pour N., Lux S.F., Paschos O., Maglia F., Lupart S., Lamp P. (2016). Inorganic solid-state electrolytes for lithium batteries: Mechanisms and properties governing ion conduction. Chem. Rev..

[24] Kalhoff J., Eshetu G.G., Bresser D., Passerini S. (2015). Safer electrolytes for lithium-ion batteries: State of the art and perspectives. ChemSusChem.

[25] Chen Y., Wen K., Chen T., Zhang X., Armand M., Chen S. (2020). Recent progress in all-solid-state lithium batteries: The emerging strategies for advanced electrolytes and their interfaces. Energy Storage Mater..

[26] Zhao Y., Ding Y., Li Y., Peng L., Byon H.R., Goodenough J.B., Yu G. (2015). A chemistry and material perspective on lithium redox flow batteries towards high-density electrical energy storage. Chem. Soc. Rev..

[27] Sakuda A., Hayashi A., Takigawa Y., Higashi K., Tatsumisago M. (2013). Evaluation of elastic modulus of Li_2_S−P_2_S_5_ glassy solid electrolyte by ultrasonic sound velocity measurement and compression test. J. Ceram. Soc. Jpn..

[28] Tatsumisago M., Takano R., Tadanaga K., Hayashi A. (2014). Preparation of Li_3_BO_3_–Li_2_SO_4_ glass–ceramic electrolytes for all-oxide lithium batteries. J. Power Sources.

[29] Sakuda A., Hayashi A., Tatsumisago M. (2013). Sulfide solid electrolyte with favorable mechanical property for all-solid-state lithium battery. Sci. Rep..

[30] Iriyama Y., Wadaguchi M., Yoshida K., Yamamoto Y., Motoyama M., Yamamoto T. (2018). 5V-class bulk-type all-solid-state rechargeable lithium batteries with electrode-solid electrolyte composite electrodes prepared by aerosol deposition. J. Power Sources.

[31] Zhu J., Li X., Wu C., Gao J., Xu H., Li Y., Guo X., Li H., Zhou W. (2021). A multilayer ceramic electrolyte for all-solid-state Li batteries. Angew. Chem. Int. Ed. Engl..

[32] Chen X., Guan Z., Chu F., Xue Z., Wu F., Yu Y. (2022). Air-stable inorganic solid-state electrolytes for high energy density lithium batteries: Challenges, strategies, and prospects. InfoMat.

[33] Wang Y., Richards W.D., Ong S.P., Miara L.J., Kim J.C., Mo Y., Ceder G. (2015). Design principles for solid-state lithium superionic conductors. Nat. Mater..

[34] Sakakura M., Mitsuishi K., Okumura T., Ishigaki N., Iriyama Y. (2022). Fabrication of oxide-based all-solid-state batteries by a sintering process based on function sharing of solid electrolytes. ACS Appl. Mater. Interfaces.

[35] Li Y., Chen X., Dolocan A., Cui Z., Xin S., Xue L., Xu H., Park K., Goodenough J.B. (2018). Garnet electrolyte with an ultralow interfacial resistance for Li-metal batteries. J. Am. Chem. Soc..

[36] Han F., Westover A.S., Yue J., Fan X., Wang F., Chi M., Leonard D.N., Dudney N.J., Wang H., Wang C. (2019). High electronic conductivity as the origin of lithium dendrite formation within solid electrolytes. Nat. Energy.

[37] Lu W., Xue M., Zhang C. (2021). Modified Li_7_La_3_Zr_2_O_12_ (LLZO) and LLZO-polymer composites for solid-state lithium batteries. Energy Storage Mater..

[38] Sun H., Kang S., Cui L. (2023). Prospects of LLZO type solid electrolyte: From material design to battery application. Chem. Eng. J..

[39] Han X., Gong Y., Fu K., He X., Hitz G.T., Dai J., Pearse A., Liu B., Wang H., Rubloff G. (2017). Negating Interfacial Impedance in Garnet-Based Solid-State Li Metal Batteries. Nat. Mater..

[40] Kim S., Kim J.-S., Miara L., Wang Y., Jung S.-K., Park S.Y., Song Z., Kim H., Badding M., Chang J. (2022). High energy and durable lithium metal batteries using garnet-type solid electrolytes with tailored lithium-metal compatibility. Nat. Commun..

[41] Zhao Y., Zheng K., Sun X. (2018). Addressing interfacial issues in liquid-based and solid-state batteries by atomic and molecular layer deposition. Joule.

[42] López-Aranguren P., Reynaud M., Głuchowski P., Bustinza A., Galceran M., López del Amo J.M., Armand M., Casas-Cabanas M. (2021). Crystalline LiPON as a bulk-type solid electrolyte. ACS Energy Lett..

[43] Hartmann P., Leichtweiss T., Busche M.R., Schneider M., Reich M., Sann J., Adelhelm P., Janek J. (2013). Degradation of NASICON-type materials in contact with lithium metal: Formation of mixed conducting interphases (MCI) on solid electrolytes. J. Phys. Chem. C.

[44] Wang S., Ding Y., Zhou G., Yu G., Manthiram A. (2016). Durability of the Li1+xTi2–xAlx(PO4)3 solid electrolyte in lithium–sulfur batteries. ACS Energy Lett..

[45] Lee R.-H., Lee D.-W., Lee J.-K., Kim K.-N., Yoon J.-R., Lee S.-H. (2024). Electrical and ionic conductivity of Li_2_O-B_2_O_3_-Al_2_O_3_ glass electrolyte for solid-state batteries. J. Energy Storage.

[46] Okumura T., Taminato S., Miyazaki Y., Kitamura M., Saito T., Takeuchi T., Kobayashi H. (2020). LISICON-based amorphous oxide for bulk-type all-solid-state lithium-ion battery. ACS Appl. Energy Mater..

[47] Dantas N.O., Silva V.A., Neto O.O.D., Nascimento M.L.F. (2012). Control of crystallization kinetics and study of the thermal, structural and morphological properties of an Li_2_O–B_2_O_3_–Al_2_O_3_ vitreous system. Braz. J. Phys..

[48] Cheng D., Wynn T.A., Wang X., Wang S., Zhang M., Shimizu R., Bai S., Nguyen H., Fang C., Kim M. (2020). Unveiling the stable nature of the solid electrolyte interphase between lithium metal and LiPON via cryogenic electron microscopy. Joule.

[49] Cheng Q., Li A., Li N., Li S., Zangiabadi A., Li T.-D., Huang W., Li A.C., Jin T., Song Q. (2019). Stabilizing solid electrolyte-anode interface in Li-metal batteries by boron nitride-based nanocomposite coating. Joule.

[50] Liu Y., Sun Q., Zhao Y., Wang B., Kaghazchi P., Adair K.R., Li R., Zhang C., Liu J., Kuo L.-Y. (2018). Stabilizing the interface of NASICON solid electrolyte against Li metal with atomic layer deposition. ACS Appl. Mater. Interfaces.

[51] Bucharsky E.C., Schell K.G., Hupfer T., Hoffmann M.J., Rohde M., Seifert H.J. (2016). Thermal properties and ionic conductivity of Li1,3Ti1,7Al0,3(PO4)3 solid electrolytes sintered by field-assisted sintering. Ionics.

[52] Moon J.I., Cho H.C., Song J.H. (2012). Synthesis and Conductive Properties of Li_1+x_Al_x_Ti_2-x_(PO_4_)_3_ (x = 0, 0.3, 0.5) by sol–gel Method. Korean J. Mater. Res..

[53] Choi S.K., Choi J., Yang M. (2023). A study on the microstructures and ionic conductivity of Li_1.3_Al_0.3_Ti_1.7_(PO_4_)_3_ with Different Synthesis Routes. J. Powder Mater..

[54] Yamada H., Morimoto N., Mukohara H., Tojo T., Yano S., Magome E., Morimura T., Bekarevich R., Mitsuishi K. (2021). Concerted influence of microstructure and adsorbed water on lithium-ion conduction of Li_1.3_Al_0.3_Ti_1.7_(PO_4_)_3_. J. Power Sources.

[55] Kang S.-G., Kim D.-H., Kim B.-J., Yoon C.-B. (2023). Sn-substituted argyrodite Li6PS5Cl solid electrolyte for improving interfacial and atmospheric stability. Materials.

[56] Meng X., Yang X.-Q., Sun X. (2012). Emerging applications of atomic layer deposition for lithium-ion battery studies. Adv. Mater..

[57] Ahmed B., Xia C., Alshareef H.N. (2016). Electrode surface engineering by atomic layer deposition: A promising pathway toward better energy storage. Nano Today.

[58] Sui D., Liu J. (2025). Constriction-Susceptible Lithium Support for Fast Cycling of Solid-State Lithium Metal Battery. Chin. Chem. Lett..

[59] Li J., Zhou M., Wu H.-H., Wang L., Zhang J., Wu N., Pan K., Liu G., Zhang Y., Han J. (2024). Machine Learning-Assisted Property Prediction of Solid-State Electrolyte. Adv. Energy Mater..

